# Percutaneous autologous bone marrow concentrate for knee osteoarthritis: patient-reported outcomes and progenitor cell content

**DOI:** 10.1007/s00264-022-05524-9

**Published:** 2022-08-06

**Authors:** Christopher J. Centeno, Dustin R. Berger, Brandon T. Money, Ehren Dodson, Christopher W. Urbanek, Neven J. Steinmetz

**Affiliations:** 1 Centeno-Schultz Clinic, Broomfield, CO USA; 2 Regenexx, Research and Development, Broomfield, CO USA

**Keywords:** Knee osteoarthritis (OA), Bone marrow concentrate (BMC), Colony-forming unit fibroblast (CFU-F), Connective tissue progenitor cells

## Abstract

**Purpose:**

Knee osteoarthritis (OA) is a common, progressively debilitating joint disease, and the intra-articular injection of autologous bone marrow concentrate (BMC) may offer a minimally invasive method of harnessing the body’s own connective tissue progenitor cells to counteract accompanying degenerative effects of the disease. However, the extent to which the progenitor cell content of BMC influences treatment outcomes is unclear. We sought to determine whether patient-reported outcome measures associated with BMC treatment for knee OA are related to the concentration of progenitor cells provided.

**Methods:**

In the present study, 65 patients (72 knees) underwent treatment for knee OA with autologous BMC and self-reported their outcomes for up to one year using follow-up questionnaires tracking function, pain, and percent improvement. A small fraction of each patient’s BMC sample was reserved for quantification with a haematological analyzer and cryopreserved for subsequent analysis of potential connective tissue progenitor cells using a colony-forming unit fibroblast (CFU-F) assay.

**Results:**

Patients reported significant increases in function and overall percent improvement in addition to decreases in pain relative to baseline levels following treatment with autologous BMC that persisted through 12 months. Patients reporting improved outcomes (46 of 72 knees) received BMC injections having higher CFU-F concentrations than non-responding patients (21.1×10^3^ ± 12.4×10^3^ vs 14.3×10^3^ ± 7.0 x10^3^ CFU-F per mL). A progenitor cell concentration of 18×10^3^ CFU-F per mL of BMC was found to best differentiate responders from non-responders.

**Conclusion:**

This study provides supportive evidence for using autologous BMC in the minimally invasive treatment of knee OA and suggests that increased progenitor cell content leads to improved treatment outcomes.

**Trial registration:**

ClinicalTrials.gov Identifier: NCT03011398, 1/7/17

**Supplementary Information:**

The online version contains supplementary material available at 10.1007/s00264-022-05524-9.

## Introduction

Osteoarthritis (OA) is a progressive joint disease that presents with chronic degeneration of articular cartilage and other deleterious bone-related changes, and conservative management of the disease is largely ineffective at reducing pain and increasing function [1]. Orthobiologic therapies have emerged as minimally invasive alternatives to traditional surgical options, largely consisting of autologous preparations derived from blood, bone marrow, or adipose tissues [2]. Bone marrow concentrate (BMC), primarily comprised of a nucleated cell-rich population (buffy coat), has been safely used to treat a variety of musculoskeletal conditions, including knee OA [3, 4]. Intra-articular BMC injections may provide improvements in both pain and function in patients suffering from symptomatic OA [5].

Autologous BMC treatment has been shown to yield better results than exercise therapy for patients with moderate to moderate–severe knee OA [6], and additional studies have reported BMC to be superior when compared with other minimally invasive treatment options, including platelet-rich plasma (PRP) and hyaluronic acid (HA) [7, 8]. Meanwhile, some have reported arthritic knees treated with BMC to have similar outcomes to those treated with HA [9] or even saline placebo [10]. Connective tissue progenitor cells, also referred to as mesenchymal stem/stromal cells (MSCs), are thought to be a key component of BMC associated with favourable clinical outcomes [11]. Positive relationships with respect to CFU-F concentration, measured using the colony-forming unit fibroblast (CFU-F) assay, and clinical outcome have been reported for orthopaedic procedures. A nearly threefold increase in CFU-F concentration was found in tibia nonunion fractures successfully treated with BMC compared to failures [12]. Similarly, patients treated with BMC for moderate to severe discogenic low back pain reported greater reductions in pain when receiving BMC containing higher concentrations of colony-forming cells [13].

Currently, the clinical importance of progenitor cells when treating knee OA with BMC is unknown. The aim of the present study was to identify individuals with moderate to severe knee OA that were treated with autologous BMC and monitored for changes in self-reported function, pain, and percent improvement up to one year post-treatment using a patient registry. Cryopreserved BMC samples were used to obtain an estimation for the progenitor cell content of the injectates used for treatment, and the resultant sample cellularity was compared with patient-reported outcomes. Other investigators have found a positive relationship between patient-reported outcomes and autologous BMC injections containing higher concentrations of progenitor cells for other orthopedic conditions [12, 13]; thus, we hypothesized a similar relationship exists for treating knee OA with BMC.

## Materials and methods

Patients treated for symptomatic knee OA at an outpatient orthopedic clinic were enrolled into an IRB approved patient registry (OHRP #IRB00002637) designed to track clinical outcomes and adverse events for autologously derived, musculoskeletal treatments. Informed consent was provided prior to entering the registry, and upon enrollment, patients were prospectively tracked using an electronic data capturing system (Dacima Software, Montreal, Quebec) that administers pre-treatment baseline and follow-up questionnaires at one, three, six, 12, and 24 months and annually thereafter. Patient-reported outcomes of interest include the Lower Extremity Functional Scale (LEFS), the Numeric Pain Scale (NPS), and a modified Single Assessment Numeric Evaluation (SANE) score (percent improvement). For the study cohort, we only utilized outcome questionnaires up to the 12-month time point.

Inclusion criteria for the present study were patients aged 35–85, having a physical examination consistent with moderate to severe knee OA, based on a Kellgren-Lawrence or Park classification grade II or greater on radiographs or MRI, respectively [14, 15], providing responses to baseline and six month and/or 12-month outcome questionnaires from the patient registry, and having a cryopreserved BMC sample available for laboratory analysis. Exclusion criteria included any knee injections within three months or knee surgery within sixmonths of the BMC treatment, the presence of inflammatory or autoimmune joint-affecting diseases, quinolone- or statin-induced myopathy/tendinopathy, current involvement in health-related litigation, condition related to worker’s compensation case, bleeding disorders, taking anticoagulants or immunosuppressive medications, and/or history of chronic opioid use.

### Processing, counting, and injection of bone marrow concentrate (BMC)

Utilizing ultrasound or fluoroscopic guidance, bone marrow was aspirated from the posterior superior iliac spine into heparinized syringes using a small-volume, multi-site technique [16, 17]. The autologous bone marrow aspirate (BMA) (60 to 120 mL in total) was manually processed into BMC (1.5 to 6.2 mL) by trained laboratory personnel, as previously detailed [18]. A component of the processing quality assurance (QA) protocol requires a small fraction of BMC (< 0.2 mL) to be set aside for cellular analysis and subsequent cryopreservation. An automated haematology analyzer (ABX Micros 60, Horiba Medical, France) was used to obtain a complete blood count for each BMC sample, and the white blood cell parameter was used as a representative measurement for the concentration of total nucleated cells [19]. The remaining portion of reserved BMC was cryopreserved at a concentration of ten million cells per mL using cryopreservation medium containing 30% fetal bovine serum (FBS) and 5% dimethyl sulfoxide (DMSO) by volume and a controlled rate freezing process [20].

All patients were encouraged to cease the use of non-steroidal anti-inflammatory drugs two weeks prior to and several weeks following BMC treatment. Under sterile conditions, patients received an intra-articular or intra-articular plus intraosseous injection(s) of BMC into the affected joint space via imaging guidance. Additionally, based on the patient’s clinical presentation and imaging findings, they may have received further injections into the supporting structures (i.e., ligaments, tendons, meniscus) if these structures were also diseased, damaged, or injured. Further, intra-articular injections of prolotherapy and concentrated leukocyte poor PRP were performed two to four days prior to (prolotherapy) and following (PRP) BMC treatment [21]. Patients with suspected ligamentous instability based on clinical indications were fitted for a hinged unilateral unloader knee brace or a patellar stabilizer brace (Breg, Inc., Carlsbad, CA, USA) and instructed to wear the brace during weight bearing activity for four weeks. If patients experienced substantial post-procedural pain, opioid rescue medication was prescribed for up to five days. Patients were advised to avoid activities that caused a worsening of pain throughout their stepwise rehabilitation protocol, which began with rest and household/community ambulation. Progression of physical activities consisted of pool or low impact exercise, followed by walking, resistance training and jogging, and ultimately advancing to full functional activity. While face-to-face physical therapy sessions were encouraged, they were not required.

### Colony-forming unit fibroblast (CFU-F) assay

Cryopreserved BMC samples were removed from cryogenic storage and rapidly warmed at 37°C for two minutes. Thawed BMC samples were promptly diluted tenfold in pre-warmed complete culture medium (CCM) containing 10% FBS and 1 ng per mL human fibroblast growth factor, counted using an automated cell counter (TC20, BioRad, Hercules, CA, USA) and trypan blue, and directly plated within standard six well tissue culture plates at two separate cell densities, 10×10^3^ and 30×10^3^ viable nucleated cells per cm^2^. Following 72 hours of culture, non-adherent cells were removed by washing, and the remaining adherent cells were maintained at 37°C and 5% CO_2_ with biweekly replacement of CCM.

After a 14-day culture period, the plates were washed and stained for colonies using crystal violet in methanol. A CFU-F was defined as any colony greater than 1 mm in diameter and containing a minimum of 100 cells. All colonies were counted by two independent observers. To determine CFU-F frequency, colony counts were averaged across wells and divided by the number of plated cells per well, and the CFU-F concentration was calculated by multiplying the CFU-F frequency of the cryopreserved sample by the nucleated cell concentration, as measured prior to cryopreservation [20].

### Statistical analysis

Patient-reported outcomes were compared over time by fitting a mixed-effects model with Geisser–Greenhouse correction and Tukey’s multiple comparison tests. Patients reporting overall favorable outcomes (responders) to autologous BMC therapy were characterized as meeting or exceeding both the minimal clinically important difference (MCID) of nine points for LEFS and 40% improvement for SANE at the 6-month follow-up (the 12-month follow-up was used for three patients not reporting 6-month functional outcomes), based on previous studies [13, 22, 23]. Patient demographics and BMC cellularity were compared between responders and non-responders using unpaired *t* tests with Welch’s correction. A receiver operating characteristics (ROC) curve analysis was used to determine the concentration of CFU-F for best differentiating responders from non-responders [24]. Reported mean outcomes of knees treated both above and below the identified target CFU-F concentration were compared against the LEFS MCID and SANE > 40% thresholds using one-sample *t* tests. Results were considered significant at *P* < 0.05. All statistical analyses were performed using GraphPad Prism 9 (GraphPad Software, La Jolla, CA, USA).

## Results

### Patient-reported outcomes following treatment with autologous BMC

**Fig. 1:**
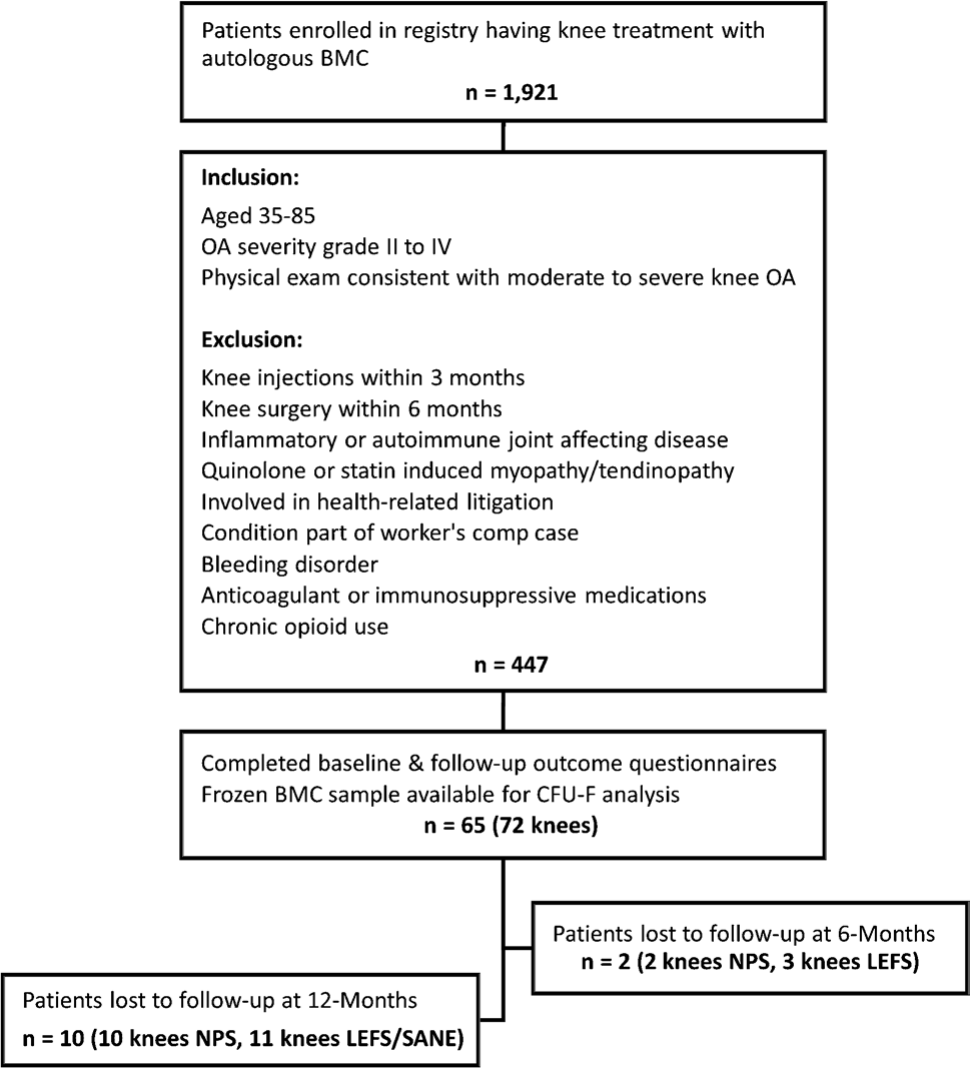
Consort flow diagram

Sixty-five patients (29 females and 36 males) aged 62.2 ± 8.2 years with moderate to severe knee OA (16.7% II, 47.2% III, 36.1% IV) were treated non-surgically using image guided, percutaneous injections of autologous BMC (Fig. 1). Seven patients were injected bilaterally for a total of 72 treated knees. Depending on the presence of bone marrow lesions on diagnostic imaging, a subset of patients (27 of 72 knees) received intra-articular as well as intraosseous BMC injections, as determined by the treating physician. An average volume of 2.9 ± 1.3 mL of BMC, containing 440 ± 155 million nucleated cells per mL, was injected into the index knee(s). The CFU-F frequency of cryopreserved BMC samples ranged from 4 to 105 CFU-Fs per million nucleated cells with an average of 43.3 ± 23.9 (0.00433% ± 0.00239%). After accounting for the range of nucleated cell concentrations measured within the BMC samples, the average concentration of colony-forming cells was 18.6×10^3^ ± 11.2×10^3^ CFU-F per mL of BMC, ranging from 925 to 57.1×10^3^. A summary of patient demographics, BMC cellularity, and reported outcomes is presented in Table 1.

**Table 1:** Summary of demographics, BMC sample composition, and patient-reported outcomes for all patients and knees

Demographics	
Patients (*n*)	65
Gender (female, male)	29F, 36M
Age (years)	62.2 ± 8.2
Body mass index (BMI)	26.9 ± 4.8
Bilateral patients (*n*)	7
Total knees treated (*n*)	72
OA severity grade	
II (%)	12 (16.7%)
III (%)	34 (47.2%)
IV (%)	26 (36.1%)
BMC composition	
BMC vol. (mL)	2.9 ± 1.3
[BMC] (×10^6^ cells / mL)	440 ± 155
CFU-F frequency (%)	0.0043 ± 0.0024
[CFU-F] (×10^3^ CFU-F/mL)	18.6 ± 11.2
Reported outcomes	
Δ NPS at 6 months (*n*)	−2.0 ± 2.0 (70)
Δ NPS at 12 months (*n*)	−2.1 ± 2.0 (62)
Δ LEFS at 6 months (*n*)	14.0 ± 13.7 (69)
Δ LEFS at 12 months (*n*)	13.6 ± 13.8 (61)
SANE at 6 months (*n*)	55.3 ± 32.1 (72)
SANE at 12 months (*n*)	55.4 ± 34.4 (61)

**Fig. 2:**
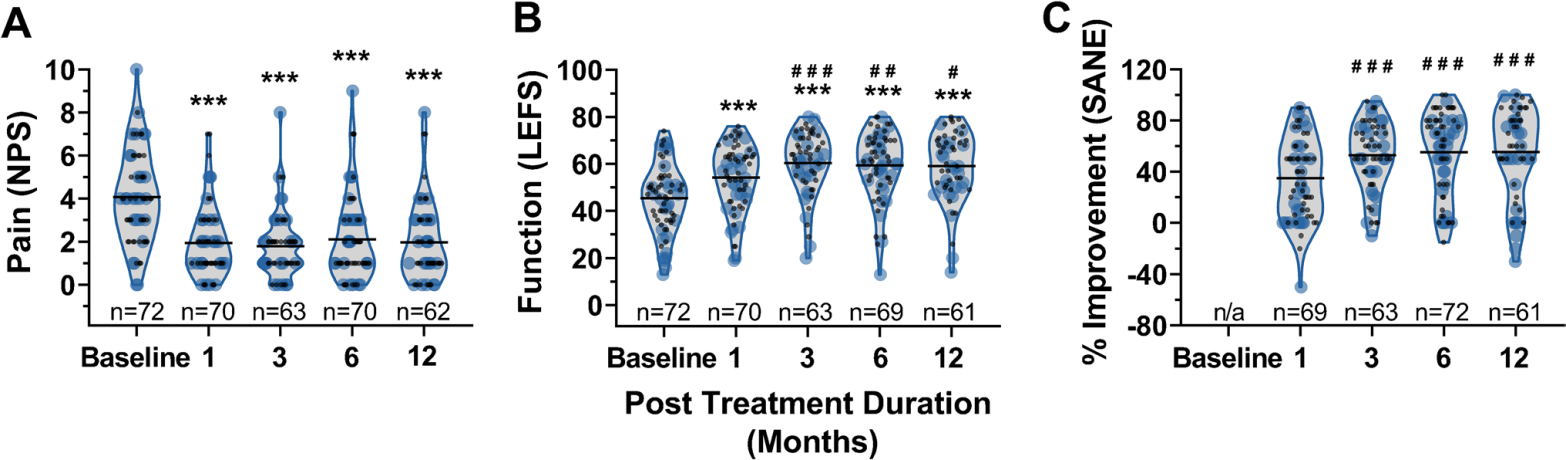
Patient-reported outcomes following treatment with autologous BMC for knee OA. Violin plots of reported **A** pain (NPS), **B** function (LEFS), and **C** percent improvement (SANE) at 1-, 3-, 6-, and 12-month follow-ups from patients receiving intra-articular (black) or intra-articular and intraosseous (blue) injections. Lines represent mean values. One symbol *P* < 0.05, two symbols *P* < 0.01, three symbols *P* < 0.001 versus baseline (*) and 1-month follow-up (^#^)

Patients treated with autologous BMC reported a considerable and sustained reduction in pain, gain in function, and overall percent perceived improvement (Fig. 2). There were no differences in reported outcomes (*P* > 0.05) between knees receiving intra-articular injections and knees receiving intra-articular plus intraosseous injections (Supplementary Fig. 1). Consequently, all outcomes were analyzed as a single cohort. Baseline pain levels (NPS) decreased from 4.1 ± 2.1 to 2.1 ± 2.0 and 2.0 ± 1.8 at the six month and 12-month follow-ups, respectively (Fig. 2A), while the knee functional scores (LEFS) increased from 45.4 ± 14.0 to 59.4 ± 14.3 and 59.1 ± 14.3 over the same period (Fig. 2B). Similarly, reported percent improvement (SANE) increased from 34.8% ± 30.9% at one month to 55.4% ± 34.4% at 12 months (Fig. 2C). Patient-reported outcomes (LEFS, NPS, SANE) were significantly improved (*P* < 0.001) over baseline (or 1 month for SANE) at all subsequent follow-ups.

While baseline and six month SANE data were collected from every patient, follow-up rates varied at other time points, ranging from 96% (69 of 72 knees) at the one month follow-up to 85% (61 of 72 knees) at the 12-month follow-up. A majority of patients (44 or 72 knees) reported in engaging in some form of post-treatment physical therapy. Moreover, ten patients reported receiving an additional injection, in the form of PRP (6), prolotherapy (2), concentrated serum proteins (1), and Hoffa’s fat pad hydrodissection (1), within the 12-month period following BMC treatment.

### Improved outcomes are reported by patients having higher CFU-F concentrations

**Fig. 3:**
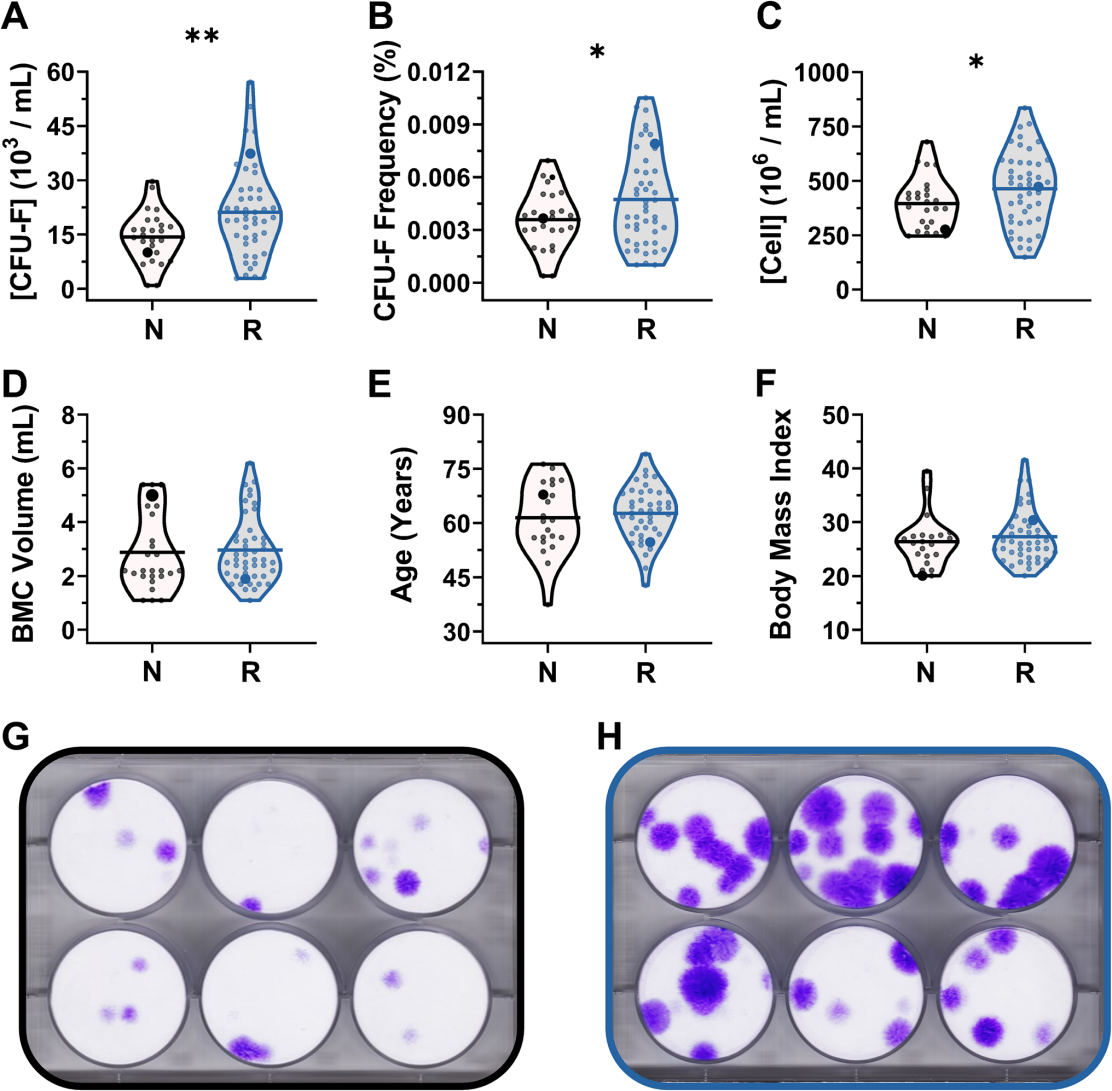
Responders to autologous BMC therapy for knee OA tend to have more progenitor cells in their injectates than non-responders. The **A** CFU-F concentration, **B** CFU-F frequency, **C** nucleated cell concentration, **D** volume, **E** age, and **F** BMI of patients and their BMC when separated based on outcome (non-responder = *N*, responder = *R*). Images of multi-well CFU-F assay plates representative of patients having (enlarged symbols in **A**–**F**) **G** lower and **H** higher CFU-F concentrations. Horizontal lines represent mean values. * *P* < 0.05, ** *P* < 0.01

A majority of patients (66%, 43 of 65) reported meeting or exceeding the LEFS MCID (9 points) and SANE threshold (> 40%) with respect to one or more treated knees at the six month follow-up and thus were considered responders. However, a subset of knees (36%, 26 of 72) failed to respond to autologous BMC treatment, and approximately one-third of the non-responding knees (9 of 26) reported deteriorating function (ΔLEFS ≤ 0) and no improvement (SANE ≤ 0) at the six month follow-up. Injectate cellularity, patient age, and BMI were compared between responding and non-responding patient cohorts (Fig. 3). The mean CFU-F concentration, frequency, and nucleated cell concentration were significantly greater (*P* = 0.004, 0.030, 0.046) within the BMC of responding knees (Fig. 3A–C), yet no differences (P > 0.05) were observed between responding knees and non-responding knees with respect to BMC volume, biological age, or BMI (Fig. 3D–F). Further, no differences in average knee OA severity grades were observed between responding and non-responding knees (*P* > 0.05). High interpatient variability in CFU-F size, density, and frequency were observed in both groups (Fig. 3G–H). A summary of patient demographics, BMC cellularity, and reported outcomes from responders and non-responders is presented in Table 2.

**Table 2:** Summary of demographics, BMC sample composition, and patient-reported outcomes when separated into responding and non-responding patient cohorts. *Reported outcomes were anticipated to be significantly different based on bifurcating the patient population based on the LEFS MCID (9) and SANE (> 40%) thresholds

	Non-responders	Responders	*P *value
Demographics			
Patients (*n*)	22	43	-
Gender (female, male)	9F, 13M	20F, 23M	-
Age (years)	61.4 ± 9.7	62.6 ± 7.5	0.622
Body mass index (BMI)	26.3 ± 4.7	27.2 ± 4.9	0.476
Bilateral patients (*n*)	3	4	-
Total knees treated (*n*)	26	46	-
OA severity grade			
OA severity grade	3.2 ± 0.7	3.2 ± 0.8	0.812
BMC composition			
BMC vol. (mL)	3.2 ± 1.5	3.0 ± 1.2	0.795
[BMC] (×10^6^ cells/mL)	395 ± 117	464 ± 169	0.046
CFU-F frequency (%)	0.0036 ± 0.0017	0.0048 ± 0.0027	0.030
[CFU-F] (×10^3^ CFU-F/mL)	14.3 ± 7.0	21.1 ± 12.4	0.004
Reported outcomes*			
Δ NPS at 6 months (*n*)	−0.7 ± 1.6 (26)	−2.7 ± 1.8 (44)	<0.001
Δ NPS at 12 months (*n*)	−1.0 ± 1.6 (19)	−2.6 ± 2.0 (43)	0.002
Δ LEFS at 6 months (*n*)	1.4 ± 7.3 (26)	21.7 ± 10.5 (43)	<0.001
Δ LEFS at 12 months (*n*)	1.1 ± 10.1 (19)	19.3 ± 11.3 (42)	<0.001
SANE at 6 months (*n*)	21.5 ± 26.7 (26)	74.3 ± 14.1 (46)	<0.001
SANE at 12 months (*n*)	23.1 ± 34.5 (18)	68.9 ± 23.9 (43)	<0.001

**Fig. 4:**
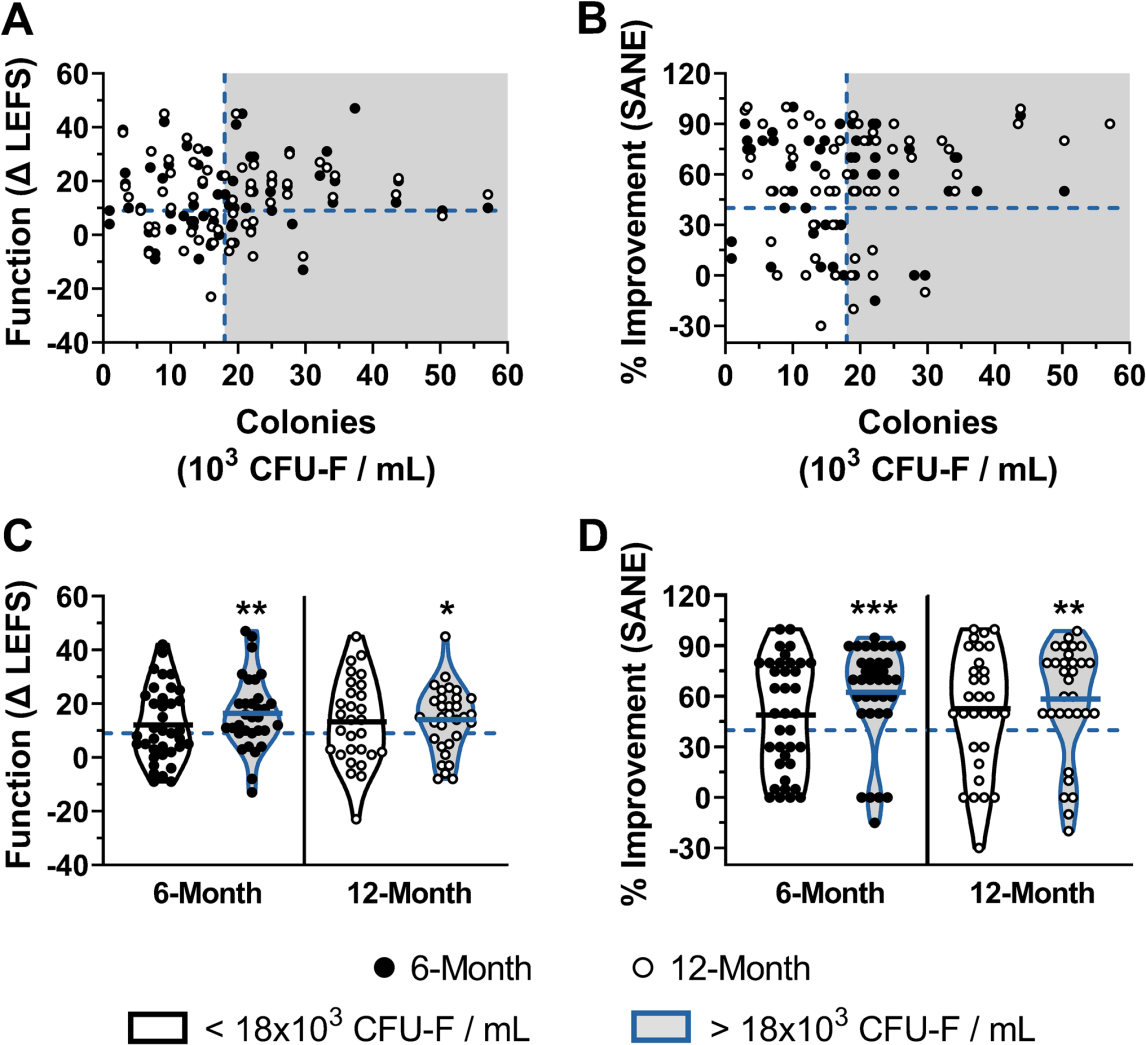
Patients having higher concentrations of CFU-F within their autologous BMC therapies generally report better outcomes. Scatter plots of **A** change in function (ΔLEFS) and **B** percent improvement (SANE) at 6-month (black symbols) and 12-month (white symbols) follow-ups versus CFU-F concentration. The **C** ΔLEFS and **D** SANE for those above and below the CFU-F concentration value of 18×10^3^ CFU-F per mL of BMC. Lines represent mean values. * *P* < 0.05, ** *P* < 0.01, *** *P* < 0.001 versus ΔLEFS = 9 and SANE = 40%

Patient-reported outcomes were compared against the CFU-F concentrations of their respective autologous BMC treatments (Fig. 4). Scatterplots of CFU-F concentration versus percent improvement and change in LEFS at both 6- and 12-month follow-up time points indicate patients having higher concentrations of colony-forming cells generally report improved outcomes (Fig. 4A–B). The receiver operating characteristic (ROC) curve (AUC = 0.677, *P* = 0.013) revealed a maximum sensitivity and specificity of 63.0% and 76.9%, respectively, at a CFU-F concentration of 18×10^3^ CFU-F per mL of BMC (Supplementary Fig. 2). When separated into two cohorts based on this CFU-F concentration cutoff, knees treated with autologous BMC having more than 18×10^3^ CFU-F per mL reported outcomes, on average, that were significantly greater than the MCID for LEFS (6-month *P* = 0.004, 12-month *P* = 0.025) and SANE (6-month *P* < 0.001, 12-month *P* = 0.005) threshold (Fig. 4C–D). In contrast, patients treated with BMC having fewer than 18×10^3^ CFU-F per mL reported average outcomes that failed to exceed the LEFS MCID and SANE threshold.

## Discussion

Patients report considerable reductions in pain, increases in function, and general overall improvement up to one year following treatment with autologous BMC for moderate to severe knee OA, consistent with our previous results and those of others [6–8, 25]. The most important finding of the present study is the increased cellularity (nucleated cell concentration, CFU-F frequency, and CFU-F concentration) measured within the BMC injectates provided to patients responding favorably compared with those that did not. Some have suggested that the progenitor cell content of BMC, as measured using a CFU-F assay, contributes to positive clinical outcomes. Patients treated with BMC injections having > 2×10^3^ CFU-F per mL reported faster and greater reductions in discogenic pain associated with lumbar degenerative disc disease [13], and a higher average CFU-F concentration, > 1.5×10^3^ CFU-F per mL, was found in the BMC of successfully treated tibial nonunion compared to treatment failure [12]. The present study determined a concentration of 18×10^3^ CFU-F per mL to best differentiate responders from non-responders for knee OA treatment.

Recently, our group detailed an alternative approach to the CFU-F assay that utilizes cryopreserved BMC in lieu of freshly obtained BMC, enabling the establishment of a biobank for the retrospective analyses of potential connective tissue progenitor cells for comparison with patient-reported outcomes [20]. To our knowledge, this is the first time the CFU-F concentration within BMC, obtained retrospectively from cryopreserved patient samples, has been reported with respect to outcomes associated with treatment for knee OA. Reported differences in CFU-F concentrations between our group and others is likely attributed to several laboratory factors. For example, BMC prepared by trained laboratory processors in our facility is three to five times more concentrated with respect to nucleated cells than BMC prepared by standard bedside devices [26]. Further, our group utilizes a low plating density to obtain the highest number of CFU-F, as recommended by others [27]. In contrast, similar studies report their plating density to be eightfold higher, which may be suboptimal for quantifying CFU-F [12, 28]. Both laboratory factors contribute to BMC with greater CFU-F concentrations than those reported by others to date.

The predictive power of the progenitor cell concentration within BMC to discriminate patient outcomes, as calculated using the area under the ROC curve is, 68%, indicating that false negative and false positive results are expected. Notably, a considerable portion of responding knees (17 of 46, 37%) were treated with BMC having fewer than 18×10^3^ CFU-F per mL (false negative). There may be factors present within BMC other than connective tissue progenitor cells, which contribute to the healing response. Elevated levels of the anti-inflammatory molecule, interleukin-1 receptor antagonist protein (IRAP), which is thought to improve OA through the inhibition of pro-inflammatory interleukin-1β (IL-1β) signaling have been found at increased levels in BMC [29]. Others have suggested the ratio of connective tissue progenitor to mononuclear cells within BMC may be another important and overlooked factor that contributes to healing [30]. In comparison, a smaller number of non-responding knees (6 of 26, 23%) received BMC injections having more than 18×10^3^ CFU-F per mL (false positive). Additional investigation is necessary to better determine patient candidacy for BMC treatment of knee OA to help exclude those patients unlikely to respond, regardless of progenitor cell content.

Being a retrospective analysis of clinical treatment registry data from a single multi-physician, interventional orthopaedic pain practice, the present study is characterized by inherent heterogeneity. Variation in patient knee OA severity, BMC volume and cellularity, primary injection type (intra-articular and/or intraosseous), unilateral vs bilateral knee treatment, whether supporting structures (i.e., ligaments, tendons, meniscus) warranted concurrent treatment, participation in post-treatment physical therapy, and receiving additional injections, among others, must be considered when interpreting the results. The present study is small and preliminary in nature; no sample size or power calculations with respect to progenitor cell content were undertaken beforehand, as currently available CFU-F data, using our laboratory approach is limited. Some patients in the cohort were lost to follow-up (up to 15% at 12 months), resulting in missing data at that time point, and while a strong correlation between fresh and cryopreserved BMC with respect to the number of CFU-F has been previously demonstrated, it is possible that the cryopreservation and cryorecovery processes adversely affect the outgrowth of some colonies [20, 31]. Additional, well-controlled prospective studies are needed and would be essential to determining how the various biological components of BMC, including colony-forming connective tissue progenitor cells, contribute to tissue healing in the context of moderate to severe OA of the knee.

In conclusion, this is the first study to retrospectively assess CFU-F concentrations from autologous BMC samples for the purpose of relating progenitor cell content with patient-reported outcomes for knee OA. Overall, responders to treatment had greater concentrations of nucleated cells and colony-forming progenitor cells. Further investigation is warranted to more accurately classify patients that will be responders to treatment based on the cellularity of their BMC.

## Supplementary Information

Supplementary Figure 1 No differences in patient reported outcomes were observed between intra-articular only and intra-articular plus intra-osseous injections of autologous BMC for knee OA (P < 0.05). Line plots of reported (**A**) pain (NPS), (**B**) function (LEFS) and (**C**) percent improvement (SANE) at 1-, 3-, 6-, and 12-month follow-ups from patients receiving intra-articular (black) or intra-articular plus intra-osseous (blue) injections. Lines represent mean values ± standard deviation. (PNG 123 kb)

High Resolution (TIF 1.70 MB)

Supplementary Figure 2 Receiver operating characteristic (ROC) curve for responders of autologous BMC therapy by CFU-F concentration (AUC = 67.7%, P < 0.05). A threshold concentration of 18×10^3^ CFU-F per mL of BMC was established by identifying the point along the ROC curve representing maximal sensitivity and specificity of 63.0% and 76.9%, respectively. (PNG 140 kb)

High Resolution Image (TIF 1.09 MB)

## Data Availability

Data may be made available upon request.
