# Supplementary figures and images for: Percutaneous autologous bone marrow concentrate for knee osteoarthritis: patient-reported outcomes and progenitor cell content
Source: Int Orthop. 2022 Aug 6;46(10):2219–28. doi: 10.1007/s00264-022-05524-9 (PMC9492580; doi:10.1007/s00264-022-05524-9)

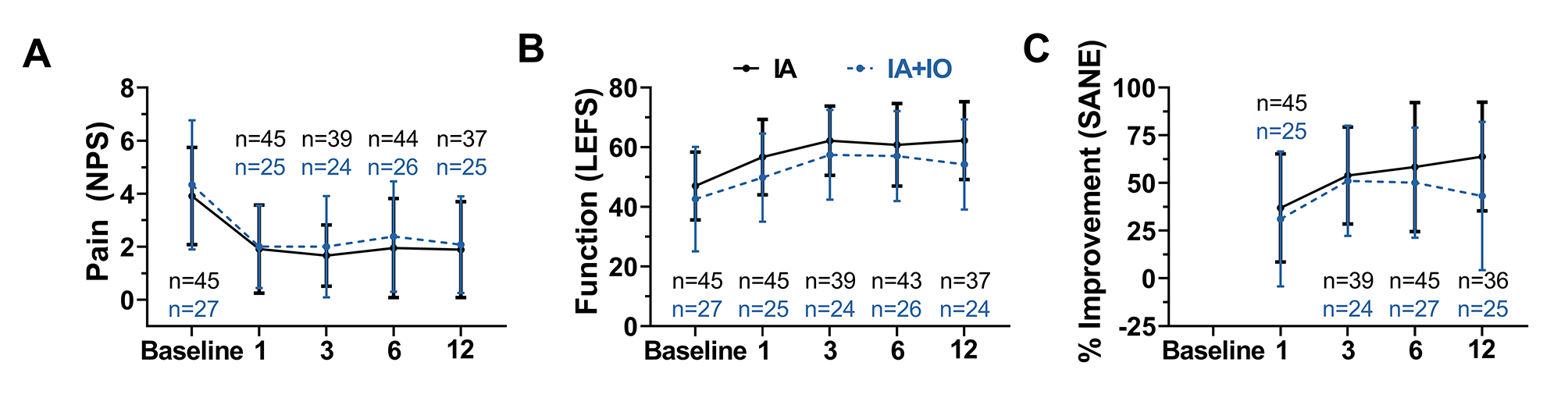

Supplement: Supplementary file 1 — No differences in patient reported outcomes were observed between intra-articular only and intra-articular plus intra-osseous injections of autologous BMC for knee OA (P < 0.05). Line plots of reported (A) pain (NPS), (B) function (LEFS) and (C) percent improvement (SANE) at 1-, 3-, 6-, and 12-month follow-ups from patients receiving intra-articular (black) or intra-articular plus intra-osseous (blue) injections. Lines represent mean values ± standard deviation. (PNG 123 kb) [file 264_2022_5524_Fig5_ESM.png]

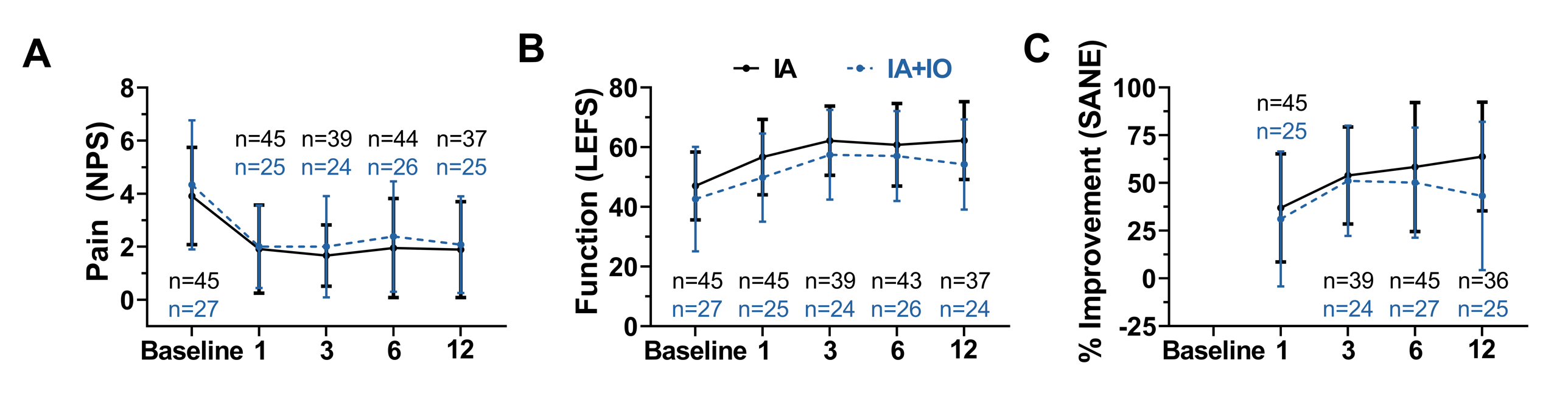

Supplement: Supplementary file 2 — High Resolution (TIF 1.70 MB) [file 264_2022_5524_MOESM1_ESM.tif]

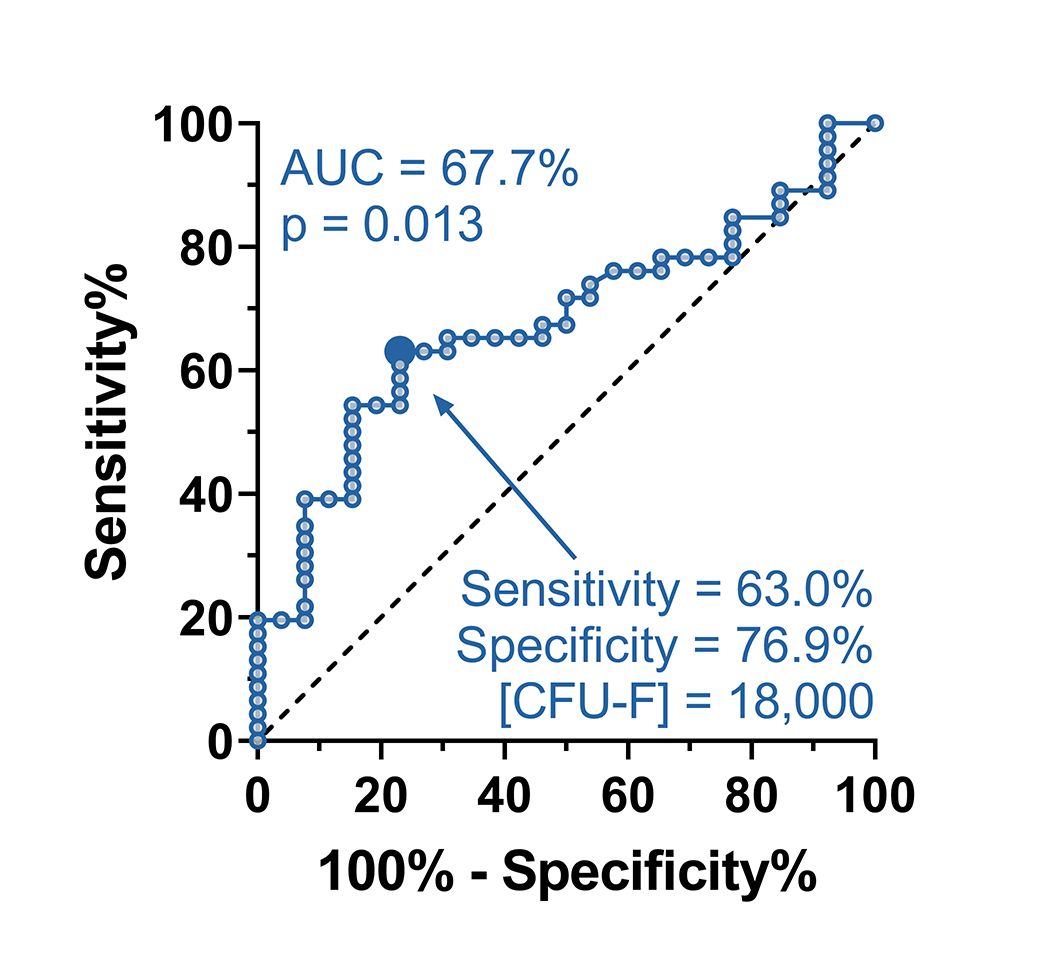

Supplement: Supplementary file 3 — Receiver operating characteristic (ROC) curve for responders of autologous BMC therapy by CFU-F concentration (AUC = 67.7%, P < 0.05). A threshold concentration of 18×103 CFU-F per mL of BMC was established by identifying the point along the ROC curve representing maximal sensitivity and specificity of 63.0% and 76.9%, respectively. (PNG 140 kb) [file 264_2022_5524_Fig6_ESM.png]

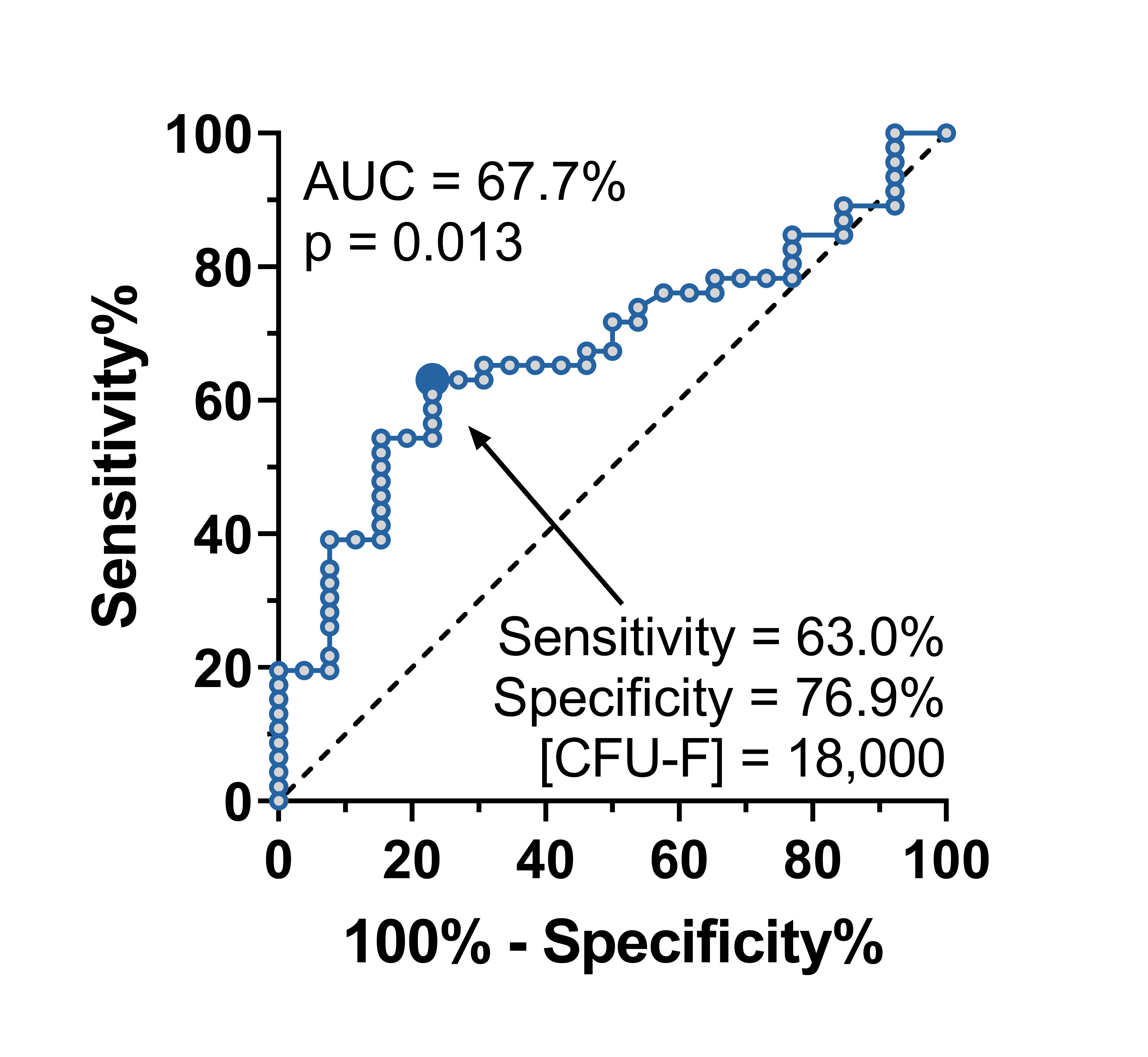

Supplement: Supplementary file 4 — High Resolution Image (TIF 1.09 MB) [file 264_2022_5524_MOESM2_ESM.tif]
